# Impact of rs599839 Polymorphism on Coronary Artery Disease Risk in Saudi Diabetic Patients

**DOI:** 10.1155/2024/8278727

**Published:** 2024-08-07

**Authors:** Neda M. Bogari, Ahmad O. Babalghith, Zohor Asaad Azher, Ahmad Hasan Mufti, Abdellatif Bouazzaoui, Hussain Banni, Abdulelah Awaji Madkhali, Ahmed Alahmadi, Reem M. Allam

**Affiliations:** ^1^ Department of Medical Genetics Faculty of Medicine Umm Al-Qura University, Makkah, Saudi Arabia; ^2^ Science and Technology Unit Umm Al Qura University, Makkah, Saudi Arabia; ^3^ Department of Pathology and Laboratory Medicine Cytogenetics Lab King Abdulaziz Medical City Ministry of National Guard Health Affairs, Jeddah, Saudi Arabia; ^4^ Department of Pathology and Laboratory Medicine King Faisal Hospital, Makkah, Saudi Arabia; ^5^ Department of Clinical Pathology Faculty of Medicine Zagazig University, Zagazig, Egypt

## Abstract

**Background:**

Coronary artery diseases may be affected by several genetic and nongenetic factors. Single-nucleotide polymorphism (SNP) rs599839 and type 2 diabetes mellitus (T2DM) can affect the occurrence and severity of coronary artery disease (CAD).

**Methods:**

Our aim was to investigate how T2DM and the rs599839 variant affected serum lipid levels and the degree of CAD patients' coronary artery stenosis. rs599839 polymorphism genotyping was done on Saudi patients with coronary angiography performed previously. Patients enrolled were divided into group A (360 DM patients), group B (225 DM patients with CAD), and group C (190 healthy volunteers as control).

**Results:**

Individuals with diabetes and CAD who possessed the GG genotype in rs599839 exhibited markedly reduced means of total cholesterol (TC), low-density lipoprotein cholesterol (LDL-C), and triglycerides (TG; 224.5, 116.2, and 221.4 versus 251.6, 131.3, and 261.7 mg/dl, *p*=0.003, 0.007, and 0.025, respectively) than AA genotype. The odds ratio and the confidence interval of 95% for G allele carriers of rs599839 were OR = 0.62, 95% CI: 0.41–0.82, and *p*=0.003, among diabetic patients with CAD.

**Conclusions:**

In patients with diabetic CAD, the locus 1p13.3 polymorphism rs599839 was found to be substantially correlated with serum lipid levels. Furthermore, among Saudi patients with diabetes, the G allele of rs599839 variant lowers the CAD risk.

## 1. Introduction

There is an overwhelming evidence that diabetes mellitus (DM) has become an epidemic on a global scale and that its prevalence is gradually increasing [1]. Cardiovascular diseases (CVD) are the leading cause of morbidity and death among diabetic patients, and type 2 diabetes mellitus (T2DM) is considered a significant independent risk factor for these conditions [2]. Furthermore, DM elevates the coronary artery disease (CAD) risk by a factor of two to six [3]. Acute thrombotic cardiovascular events are the primary cause of mortality in CAD [4]. Moreover, a number of other established risk factors for CAD exist, including genetics, smoking, hypertension, dyslipidemia, obesity, and sedentary lifestyle [5].

Genome-wide association studies (GWAS) have recently shown that a number of genetic loci represent risk or protective factors of CVD, and these loci are associated with multiple single-nucleotide polymorphisms (SNPs) mapped at 1p13.1, whereas including this region, five genes; cadherin, myosin-binding protein H-like (MYBPHL), proline-serine-rich coiled-coil 1 (PSRC1), sortilin 1 (SORT1), and EGF LAG Seven-Pass G type receptor 2 (CELSR2) [6].

Sortilin 1 protein, encoded by the SORT1 gene, is engaged in a number of lipid-associated processes, including the secretion of very low density lipoprotein-cholesterol (VLDL-C), the formation of atherosclerotic plaques, the metabolism of LDL cholesterol, and the secretion of PCSK9 [7]. It also contributes to other pathophysiological processes such as insulin resistance, vascular calcification, inflammation, dyslipidemia, and foam cell formation [8]. The rs599839 SNP locates between PSRC1 and CELSR2 genes at a noncoding region [9].

Saudi healthcare resources are heavily burdened by the increasing frequency of diabetes and CAD. For better understanding the role of rs599839 and assess its potential to either induce or protect against CAD, this study sets out to investigate the relationship between rs599839 and the risk, severity, and eventual clinical outcomes of CAD in type 2 diabetes.

## 2. Subjects and Methods

### 2.1. Participants

A number of 585 T2DM patients with diagnoses made in accordance with ADA2017 participated in a cross-sectional study, while 190 healthy volunteers served as the control group who, based on their age and sex, were matched with cases. The study participants underwent elective coronary angiography for suspected CAD. Diabetic patients were further subdivided angiographically into two groups, group A: 360 patients (CAD-free), group B: 225 diabetic patients with CAD, while group C: The control group is CAD-free. Patients were recruited at Al-Noor specialized hospitals as well as King Abdullah Medical City (KAMC) in Mecca from March 1, 2019, to April 31, 2021.

A statistical specialist used the open Epi version 6 program to determine the sample size at a 95% confidence level and 80% study power [10], using the prevalence of CAD and DM announced by the Ministry of Health (MOH) Yearbook of Statistics, and the frequency of the mutant genotype in Gulf nations was found to be around 47.4% [11].

We excluded individuals diagnosed with decompensated heart failure, rheumatic valvular heart diseases, diabetes mellitus type 1 (T1DM), patients with eGFR lower than 60 ml/min/1.73 m^2^, or patients with decompensated liver disease and prior myocardial infarction from this study.

Participants underwent a comprehensive clinical evaluation and took their history in great detail. Measurements of weight, height, hips, and waist were obtained. Systolic and diastolic blood pressure (SBP and DBP) were recorded. Weight (in kg)/height (in m^2^) was used to calculate BMI. The use of existing heart, lipid-lowering, and antidiabetic medications was documented.

### 2.2. SYNTAX Score

An angiographic grading system called the SYNTAX score is a tool for assessing the complexity of CAD. It represents the total points allotted to each lesion found in the coronary artery tree that narrows more than 50% of the vessel's diameter, when the diameter is greater than 1.5 mm. Depending on the degree of disease present, each segment receives a score of 1 or 2. After that, this score is then weighted using a chart, with values ranging from 0.5 for smaller branches to 5.0 for the left main and 3.5 for the proximal left anterior descending artery (LAD). Points are added for further description of the lesions. Next, each of these features is added up by the SYNTAX score algorithm to get the overall SYNTAX score [12]. An algorithm for SYNTAX scores was used to determine the overall SYNTAX score available on the SYNTAX website (http://www.syntaxscore.com). Our study protocol was approved by the Umm Al-Qura University Ethical Committee, and an informed consent form was signed by each participant.

### 2.3. Sample Collection and Biochemical Assay

Following an overnight fast, the subjects had venous blood samples drawn to measure triglycerides (TG), high-density lipoprotein cholesterol (HDL-C), low-density lipoprotein cholesterol (LDL-C), total cholesterol (TC), fasting serum insulin (FSI), and fasting and postprandial blood glucose (Cobas, Roche Diagnostics, Mannheim, Germany). Additionally, a whole blood sample obtained via venous EDTA was tested for HbA1c using the colorimetric method. The following formula: (fasting plasma glucose (FPG; mg/dl) × FSI (mU/ml)/405), was made for calculation of the homeostasis model assessments of insulin resistance (HOMA-IR) according to our earlier protocol [13].

### 2.4. Molecular Analysis

As the manufacturer recommends, a whole blood sample was used for DNA extraction (ThermoFisher Scientific, Waltham, MA, USA). Testing for DNA quality was done with the NanoDrop2000 device (ThermoFisher Scientific, Wilmington, DE, USA) for the detection of optical density values and 1.25% agarose gel electrophoresis. Using 100 ng of genomic DNA and probes of allele-specific TaqMan genotyping technique (Applied Biosystems, Foster City, California, USA), participants were genotyped for rs599839.

Using the StepOne real time PCR system, the genotyping and PCR amplification were carried out (ThermoFisher Scientific, Wilmington, DE, USA) by relying on default conditions as optimized by the supplier company. Reaction mix per well was as follow: 2 *µ*l of genomic DNA was added to master mix consisting of 0.5 *µ*l TaqMan 40X Assay mix (probes and primers), 10 *µ*l TaqMan Genotyping master mix, and 7.5 *µ*l nuclease-free water to obtain totally 20 *µ*l reaction mix. After covering the plate via transparent adhesive film and momentary spin, the plate was immediately inserted in the machine to start the run and get results after 90 min.

### 2.5. Statistical Analysis

We used SPSS version 21 for all statistical analyses (Chicago, IL, USA). We represented quantitative variables as the median ± interquartile range (IQR) or the mean ± standard deviation, but we used percentages for expression of qualitative variables. The *χ*^2^ test was used to look at gender, smoking status, hypertension, and the SNP site. The independent samples *t* test was used to examine the serum level of the quantitative variables, and the Mann–Whitney test was used to examine the remaining baseline characteristics related to the study population's demographics. We used one-way ANOVA test to compare the different genotypes regarding the quantitative variables, while the remaining baseline characteristics were tested by the Kruskal–Wallis test. The alleles distribution between the two groups was tested by Fisher's exact test. The association significance between CAD and SNP rs599839 was performed by logistic regression analysis. Additionally, we computed odds ratios (OR) and the 95% confidence intervals (CI). The Hardy–Weinberg law examines the balance of allele and genotype frequencies in two groups. A statistically significant difference between the two groups was defined as *p*  < 0.05.

## 3. Results

The control group's mean age was 60.3 ± 6.3 years, with 62.1% of the participants being male. The mean age of the diabetic group (group A) was 59.3 ± 5.8 years, with 61.1% of the participants being male. The diabetic group with CAD (group B) had mean age of 61.4 ± 7.4 years, and 57.3% of the participants were men. Age, ethnicity, and sex were matched between the control and T2DM groups.

### 3.1. Anthropometric and Laboratory Characteristics

When compared to the control group, the lipid profile (TG, TC, and LDL-C) and SBP and DBP values of group A were significantly higher. Additionally, the fasting glucose, insulin, HbA1c, and HOMA-IR values of the diabetic patients were significantly higher when compared to those of the control group. In contrast, patients with type 2 diabetes had lower HDL-C levels in comparison to those in the control group (Table 1).

Diabetic patients with CAD (group B), when compared to the diabetic group (group A), had significantly higher serum lipids and values of fasting glucose, FSI, HbA1c, and HOMA-IR. Furthermore, when compared to group A, group B had substantially longer durations of diabetes, smoking, and sedentary lifestyle.

### 3.2. CAD Severity by SYNTAX Score

**Figure 1:**
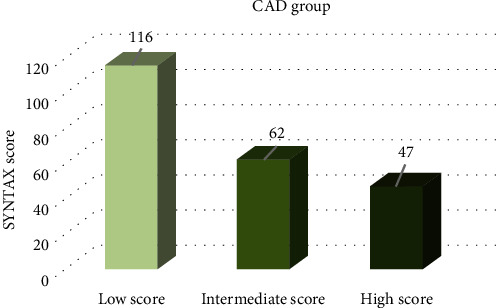
Estimated SYNTAX score in diabetic patients with CAD, evaluating the degree of CAD based on results from coronary angiography. A number of 47 patients showed a high score (≥33), 62 patients revealed an moderate score (23–32), and 116 patients showed a low score (0–22).

Applying SYNTAX score, and based on coronary angiography results, CAD severity was assessed. In patients with diabetes, the SYNTAX score as mean ± standard deviation were 21.8 ± 2 which was significantly lower than group of diabetes with CAD (28.3 ± 2.9, *p*=0.002). Forty-seven patients had a high score (≥33) and 62 patients showed an moderate score (23–32), while 116 patients revealed a low score (0–22; Figure 1).

### 3.3. Genotype and Allele Frequencies

With a *p* value > 0.05, the genotype frequency was complied with the Hardy–Weinberg equilibrium (HWE).  Table 2 displays the rs599839 polymorphism genotype distribution for each subject. Across all research participants, the AA genotype for rs599839 was more common (70.8% for group A and 75.6% for group B, respectively). The percentage of minor G allele for rs599839 was 18.1% and 14.7% in groups A and B, respectively. The *χ*^2^ test proved that the presence of at least one minor G allele in the dominant genetic model plays a protective role in diabetic patients with CAD (OR = 0.62, 95% CI: 0.41–0.82, and *p*=0.003). G allele when compared to A allele separately, showed a protective role in CAD diabetic patients (OR = 0.64, 95% CI: 0.47–0.86, and *p*=0.006; Table 2).

### 3.4. Genotypes Association with Lipid Parameters

Serum lipid concentrations were assayed according to the rs599839 genotypes and were adjusted for hypertension, smoking, BMI, and antidiabetic and statin therapy.  Table 3 demonstrates that the GG genotype in diabetic patients with CAD was associated with substantially lower means of TC, TG, and LDL-C levels than AA genotype (224.5, 116.2, and 214.2 versus 251.6, 131.3, and 261.7 mg/dl, *p*=0.003, 0.007, and 0.025, respectively) and higher mean HDL-C (43.4 versus 24.6 mg/dl, *p*=0.026). The minor G allele of rs599839 was linked to significantly lower means of TC, TG, and LDL-C levels as well as higher HDL-C, when compared to the A allele.

**Figure 2:**
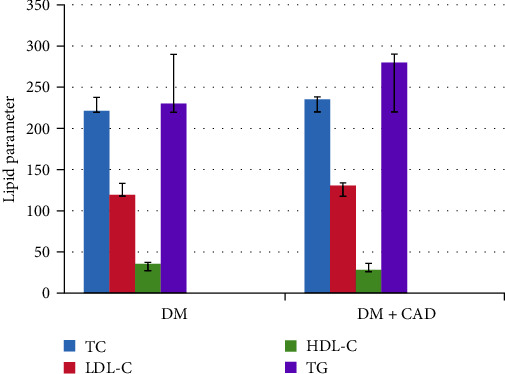
Estimated lipid parameters in patients groups. Group B with CAD had significantly higher means of TC, TG, and LDL-C levels and lower HDL-C level than group A.

Lipid parameters have been compared between group A and group B. It was found that group B (diabetic patients with CAD) had significantly higher means of TC, TG, and LDL-C levels and lower HDL-C level than group A (diabetic without CAD; Figure 2).

### 3.5. Relationships between Study Participants' Genotype and Cardiac Risk Factors

Additionally, we measure how the genotype and CAD risks affect the SYNTAX score that represents the severity of the disease. Regression analysis was used to separate the effects of rs599839 variant, age, gender, BMI, lipid parameters, duration of diabetes, hypertension, and other independent variables on SYNTAX score as the dependent factor in all CAD patients. As indicated in Table 4, the findings demonstrate that five of the independent variables have a statistically significant impact on the SYNTAX score: TC (*p*=0.002), LDL-C (*p*=0.008), TG (*p*=0.005), DM duration (*p*=0.025), and rs599839 (*p*=0.015).

## 4. Discussion

DM (T2DM) is the most frequent cause of morbidity and death in patients with diabetes and a significant independent risk factor for CAD. Subjects with type 2 diabetes had a higher risk of heart failure by 112%, 53% of MI, 58% of stroke, and 10% higher risk of CAD. T2DM is thus a significant risk factor for CVD and its effects [14].

Improved knowledge of the etiology of T2DM and CVD is crucial for better clinical management, early patient identification and prediction, and identification of at-risk individuals. Our study aimed to explore the function of rs599839, assessing how this SNP may either induce or protect against CAD, and assessing the relationship between rs599839 and the risk, severity, and eventual clinical consequences of CAD in type 2 diabetes.

Our findings showed that in diabetic patients, the minor G allele of the variant rs599839 exerted a protective role against CAD in patients with T2DM (OR = 0.62, 95% CI: 0.41–0.82, and *p*=0.003). The genotypic frequencies of rs599839 decreased in the dominant model in patients with T2DM and CAD, which revealed that rs599839 AG + GG were accompanied by a decreased risk of CAD in patients with T2DM (OR = 0.64, 95% CI: 0.47–0.86, and *p*=0.003). The rs599839 minor G allele was linked to significant lower means of TC, TG, and LDL-C levels and higher HDL-C than A allele.

Maching with our findings, Arvind et al. [9] discovered that in Indians, the minor G allele has been linked to decreased levels of TC and LDL-C and a decreased risk against CAD (OR = 0.422 and 95% CI: 0.181–0.981). The protective role of minor G allele was proven through several genetic models in a Mexican population (OR additive model = 0.72, 95% CI: 0.56–0.92, and *p*=0.009 and OR dominant model = 0.66, 95% CI: 0.49–0.89, and *p*=0.007) [15].

Also, it was discovered to decrease serum levels of LDL-C in Japanese population (OR = 0.7 and 95% CI: 0.57–0.85) [16] and to decrease coronary stenosis risk in Arab CAD individuals (OR = 0.51 and 95% CI: 0.30–0.92) [17].

In meta-analyses, it has been found that the major A allele of rs599839 elevates the CAD risk in Asians (OR = 1.31 and 95% CI: 1.17–1.47) [18] and Europeans (OR = 1.11 and 95% CI: 1.08–1.15) [19].

Han et al. [20] reported higher risk of CAD for A allele in the Chinese population when patients and healthy controls were compared (OR = 8.37 and 95% CI: 1.70–41.0) [20].

Given that rs599839 is located on 1p13.3 chromosomal region, which has been connected to LDL-C in multiple recent GWAS, it may have a cardioprotective function [21, 22, 23, 24, 25, 26] and harbors four genes: PSRC1, CELSR2, MYBPHL, and sortilin 1 (SORT1).

It has been proven that SORT1 controlled the intracellular breakdown and endocytosis of lipoprotein lipase (LPL), which limits the rate with which TGs are hydrolyzed in lipoproteins [27]. Recently, sortilin has also been connected to the apoA-V endocytosis with its chylomicrons content [28].

Both TG metabolism and LDL-C metabolism were found to be significantly correlated with the rs599839 polymorphism by Kleber et al. [29]. They verified that the rs599839 polymorphism is linked to CAD and MI by observing a correlation between the radius of LDL-C particles and the genotype.

## 5. Conclusion and Recommendations

Our study highlighted the role of rs599839 polymorphism in diabetic patients when comparing group of diabetic patients with another diabetic group with CAD diagnosed angiographically. We proved the cardioprotective and lipid-lowering effect of rs599839 polymorphism in diabetic patients.

## 6. Strengths and Limitations

This study, as far as we know, is the first of its kind in Saudi Arabia to correlate the rs599839 polymorphism to T2DM and CAD. G allele of rs599839 polymorphism may alleviate the CAD risk in patients with T2DM which itself is an independent risk factor for CAD. Tight glycaemic control in diabetic patients having A allele is a must.

However, our study does have some limitations. First, we have a limited sample size, this may hinder results generalisation across the whole country. Larger cohort studies are needed. Additionally, participants were enlisted from two hospitals located in Saudi Arabia's western region. Selection bias would also have been difficult to avoid in a multicenter study.

## Figures and Tables

**Table 1:** Baseline demographic and biochemical characteristics of groups under study.

Variable	Group A DM (360)	Group B DM + CAD (225)	Group C healthy volunteers (190)
Age	59.3 (5.8)	61.4 (7.4)	60.3 (6.3)
Gender (male *n*, %)	289 (61.1%)	182 (57.3%)	118 (62.1%)
BMI (kg/m^2^)	29.3 (3.1) ^*∗*^	30.2 (3.3) ^*∗*^	25.3 (2.6)
Body weight (kg)	81.1 (4.3) ^*∗*^	85 (5.8) ^*∗*^	74.2 (2.6)
Waist circumference (cm)	82.1 (5.4) ^*∗*^	102.3 (4.1) ^*∗*^	81.4 (3.1)
Smoking (%)	108 (30%) ^*∗∗*^	101 (44.9%) ^*∗∗*^	22 (11.6%)
Sedentary life (%)	120 (33.3%) ^*∗∗*^	120 (53.3%) ^*∗∗*^	30 (15.8%)
Systolic BP (mmHg)	126.7 (4.7) ^*∗*^	138.5 (6.7) ^*∗*^	118.2 (3.7)
Diastolic BP (mmHg)	88.5 (3.6) ^*∗*^	92.3 (4.1) ^*∗*^	76.4 (2.9)
Fasting glucose (mg/dl)	126.7 (6.9) ^*∗*^	135.3 (4.2) ^*∗∗*^	92.3 (5.1)
Fasting insulin (lU/ml)	7.52 (1.8) ^*∗*^	25.58 (11.6) ^*∗*^	8.945 (1.07)
HbA1c (%)	9.44 (1.89) ^*∗*^	10.8 (0.95) ^*∗∗*^	4.8 (0.23)
HOMA-IR	1.84 (0.9) ^*∗*^	7.75 (5.7) ^*∗∗*^	1.8 (0.35)
DM duration (years)	12.4 (5.3)	20.23 (5.9) ^*∗∗*^	—
Total cholesterol (mg/dl)	223.1 (14.6) ^*∗∗*^	235.6 (19.5) ^*∗*^	151.67 (12.4)
HDL-C (mg/dl)	35.2 (8.1) ^*∗∗*^	28.3 (9.3) ^*∗∗*^	39.6 (10.6)
LDL-C (mg/dl)	120.7 (21.4) ^*∗∗*^	131.3 (25.3) ^*∗∗*^	93.6 (12.6)
Triglycerides (mg/dl)	231.1 (17.2) ^*∗∗*^	280.3 (14.5) ^*∗*^	182.1 (13.3)
SYNTAX score	21.8 (2.6) ^*∗∗*^	28.3 (2.9) ^*∗*^	17 (1.4)
DM treatment			
Oral drugs	101 (28.1%)	85 (37.8%) ^*∗*^	—
Insulin	43 (11.9%)	37 (16.4%)	—
Mixed	33 (9.2%)	25 (11.1%) ^*∗*^	—
Cardiac medication			
* β*-Blocker	94 (26.1%)	88 (39.1%) ^*∗∗*^	—
ACEI/ARB	115 (31.9%)	92 (40.8%) ^*∗∗*^	—
Statin	121 (33.6%)	96 (42.7%) ^*∗∗*^	—

CAD, coronary artery disease; FSI, fasting serum insulin; HDL-C, high-density lipoprotein cholesterol; HOMA-IR, homeostasis model assessments of insulin resistance; LDL-C, low-density lipoprotein cholesterol; T2DM, type 2 diabetes mellitus; and ACEI/ARB, angiotensin-converting enzyme inhibitor/angiotensin receptor blocker. Student *t* test and *χ*^2^ test between groups A against B and A against C.  ^*∗*^*p*  < 0 : 01,  ^*∗∗*^*p*  < 0 : 001.

**Table 2:** Genotypes and alleles frequencies among study subjects.

Genotype	Group A DM (360; %)	Group B DM + CAD (225; %)	OR (95% CI)	*p*
AA	255 (70.8)	170 (75.6)	1	—

AG	80 (22.3)	44 (19.6)	0.82 (0.54–1.72)	0.314

GG	25 (6.9)	11 (4.8)	0.58 (0.27–0.84)	**0.002**

Dominant model				
AG + GG	105 (29.2)	55 (24.4)	0.62 (0.41–0.82)	**0.003**

Recessive model				
AA + AG	335 (93.1)	214 (95.1)	1.4 (1.06–1.92)	0.613

Allele				
A	590 (81.9)	384 (85.3)	1	**0.006**
G	130 (18.1)	66 (14.7)	0.64 (0.47–0.86)	

Bold values indicates a statistically significant difference.

**Table 3:** Lipid profile estimated according to the genotypes of the studied patients.

Genotypes	TC	LDL-C	HDL-C	TG
AA	251.6 ± 15.3	131.3 ± 8.6	24.6 ± 4.6	261.7 ± 15.9
GA	231.1 ± 14.5	122.3 ± 6.4	32.35 ± 8.1	233.6 ± 21.1
GG	224.5 ± 12.3 ^*∗∗*^	116.2 ± 4.6 ^*∗∗*^	43.4 ± 6.5 ^*∗∗*^	221.4 ± 12.9 ^*∗*^
G allele	221.4 ± 10.3 ^*∗∗*^	110 ± 3.5 ^*∗*^	44.2 ± 5.1 ^*∗*^	214.2 ± 10.5 ^*∗*^

^*∗*^ANOVA test *p* value < 0.05  ^*∗∗*^ *p* value < 0.001.

**Table 4:** Analysis of regression for related risk factors.

Variable	Unstandardized coefficients		Standardized coefficients	
	*B*	SE	*β*	*p*
Age	0.612	0.027	0.018	0.327
Gender	0.524	1.432	0.650	0.266
BMI	0.618	0.129	0.18	0.093
TC	0.061	0.641	1.92	**0.002**
LDL-C	0.033	0.214	0.22	**0.008**
TG	0.021	0.816	1.89	**0.005**
DM duration	0.352	0.411	0.38	**0.025**
HPN	0.378	0.316	0.19	0.060
rs599839	−0.231	0.712	−0.09	**0.015**

At levels of *β*-coefficient at two-tailed testing *p*  < 0.05 is significant. Bold values indicates a statistically significant difference.

## Data Availability

Upon reasonable request, the corresponding author can supply the datasets utilized and/or analyzed in the current work.
